# Preclinical validation of a novel compound targeting p70S6 kinase in breast cancer

**DOI:** 10.18632/aging.100954

**Published:** 2016-05-04

**Authors:** Ilenia Segatto, Samuele Massarut, Robert Boyle, Gustavo Baldassarre, David Walker, Barbara Belletti

**Affiliations:** ^1^ Division of Molecular Oncology, CRO, National Cancer Institute, Aviano 33081, Italy; ^2^ Breast Surgery Unit, CRO, National Cancer Institute, Aviano 33081, Italy; ^3^ Sentinel Oncology Limited, Cambridge, United Kingdom

**Keywords:** breast cancer, p70S6K, inhibitor, signaling pathway, survival, proliferation, recurrence

## Abstract

Breast cancer is a frequent and treatable disease. However, when recurrent, breast cancer often becomes refractory to therapy and progresses into metastatic forms that are typically incurable. Thus, understanding and targeting the critical pathways underlying breast cancer recurrence is urgently needed to eradicate primary disease and achieve better prognosis. Recently, we have demonstrated that the ribosomal protein p70S6K is activated in residual breast cancer cells as a result of post-surgical inflammation and that interfering with its activity in the peri-operative setting strongly suppresses recurrence in a mouse model. In order to develop clinically-exploitable treatments targeting p70S6K, we have tested a newly generated compound, called FS-115. FS-115 potently inhibited p70S6K1 (IC_50_ 35nM) with high selectivity over other AGC kinases or PI3K pathway kinases. *In vitro*, treatment with FS-115 efficiently blocked p70S6K activity in breast cancer cell lines and impaired colony formation and anchorage independent growth. Pharmacokinetic profiling showed that FS-115 exhibited high oral bioavailability, optimal plasma distribution and high brain penetrance. In nude mice, FS-115 strongly suppressed tumor take-rate and primary tumor growth. Oral dosing with FS-115 in a peri-operative schedule was effective in decreasing local recurrence of breast cancer and a long-term treatment schedule was well tolerated and efficiently suppressed distant metastasis formation. Altogether, we propose that FS-115 might be a good candidate for the treatment of breast cancer patients at high risk to relapse.

**Summary Statement:**

Our results confirm that inhibition of p70S6K represents a valuable opportunity for restraining loco-regional relapse and metastasis in breast cancer and identify in FS-115 a promising candidate-inhibitor to move from preclinical to clinical treatments.

## INTRODUCTION

The 70 kDa ribosomal protein S6 kinase (hereafter p70S6K) is a serine/threonine kinase regulated by the phosphoinositide 3-kinase (PI3K)/mammalian target of rapamycin (mTOR) pathway [1]. p70S6K can be regulated *via* different mechanisms, primarily involving protein phosphorylation, dephosphorylation, ubiquitination, acetylation and also proteolytic cleavage. Following growth factor or hormones stimulation, or nutrient inputs, the PI3K/mTOR pathway is activated, crucially integrating extra- and intra-cellular signals thereby finely regulating cell survival, growth and metabolism, inducing numerous anabolic processes, including protein and lipid synthesis [2]. Upon activation, the mTOR kinase, when part of mTOR complex 1 (mTORC1) phosphorylates, among other substrates, p70S6K, which, in turn, phosphorylates S6 protein of ribosomal subunit 40S. S6 phosphorylation results in selective translation of unique family of mRNAs (5′TOP), coding for the components of the translational apparatus [3].

Given the widespread deregulation of the PI3K/mTOR pathway in human tumors, as well as in other pathological conditions, p70S6K has been largely used as biomarker for response to inhibitors of the mTOR (rapamycin analogues). However, this is not ideal as mTORC1 also phosphorylates other substrates. Inhibition of mTOR has so far achieved limited clinical success, due to high toxicity and modest clinical improvements. It has been proposed that the efficacy of these inhibitors may be limited by feedback activation of the pathway in response to mTOR inhibition, particularly *via* AKT [4]. However, whether this feedback activation is predictive of a loss of clinical response is not completely clear [5,6].

Due to its specific role in controlling protein synthesis and also its involvement in a variety of human diseases ranging from diabetes and obesity to cancer, p70S6K is being considered as a promising therapeutic target for drug development. Suppressing the activity of p70S6K is predicted to inhibit ribosome biogenesis and synthesis of angiogenic and cell-cycle regulatory proteins [7].

For these reasons, novel inhibitors of p70S6K have been generated, displaying specificity for p70S6K, for both biochemical studies and clinical applications. PF-4708671 was the first specific p70S6K1 isoform inhibitor to be reported [8] and has served for many biochemical and preclinical studies. Its use *in vitro* and *in vivo*, to block tumor cell growth and relapse, displayed biological effects superior to those elicited by mTOR inhibitors, at least in the context of breast cancer [9,10]. Recently, a novel inhibitor against p70S6K activity, LY2584702 tosylate, alone or in combination with erlotinib or everolimus, was tested in phase I trials, in patients with advanced solid tumors with very limited clinical benefit [11,12]. Part of the failure dealt with its bad performance in pharmacokinetic analyses and in the low tolerability, particularly when administered with erlotinib. PI3K/mTOR pathway aberrations are common in breast cancer and associated with resistance to endocrine and human epidermal growth factor receptor 2 (HER2) targeted therapies. Thus, more recent trials are evaluating mTOR inhibition with everolimus in the adjuvant setting in high-risk estrogen receptor-positive, HER2-negative early breast cancer (BOLERO-2) [13,14] or in trastuzumab resistant, HER-2 positive, advanced breast cancer patients (BOLERO-3) [15]. Results from these trials were more encouraging, at least in terms of progression free survival (not significant for overall survival), although issues related to the urgent need of biomarkers for more precise patient stratification and still elevated toxicities, are limiting the enthusiasm. The centrality of mTOR as a node at which growth factors, hormones and nutrient inputs converge, in normal as well as in cancer cells, makes of it the most promising and, at the same time, the weakest of the possible targets.

Many data suggest that p70S6K itself is implicated in breast cancer onset and progression. The chromosomal region 17q23 containing the p70S6K gene (RPS6KB1) is amplified in approximately 10% of all primary breast cancer cases, leading to p70S6K overexpression [16–19] and this amplification is associated with poor prognosis [16,20–22]. Interestingly, overexpression of p70S6K protein is linked to increased risk of locoregional recurrence in node-negative early breast cancer patients [20]. Moreover, p70S6K is highly sensitive to signals coming from growth factors, amino acids, nutrients and hypoxia and relays these signals to regulate a vast list of substrates, eventually controlling many oncogenic processes, such as cell growth and proliferation, survival and apoptosis, autophagy, migration and invasion [1,23]. Our previous work uncovered that p70S6K activity is robustly induced by surgery in breast cancer patients and that its inhibition, using PF-4708671, strongly impaired breast cancer local relapse in a mouse model of breast cancer, supporting the possibility that it may be crucial also for breast cancer recurrence in human patients [9,10,24]. Several clinical strategies are currently available to restrain recurrence in breast cancer patients, including various surgical and radiotherapy options. These clinical decisions have a significant impact on treatment-related morbidity and disease free- and overall-survival of patients. Here, we pursue the goal of moving from preclinical into clinically-exploitable treatments and characterize the action of a novel selective compound targeting p70S6K. Our results confirm that inhibition of p70S6K activity may represent a valuable opportunity to restrain loco-regional and distant relapse in breast cancer patients.

## RESULTS

### Generation of FS-115, a potent, selective and orally bioavailable inhibitor of p70S6K1

**Figure 1:**
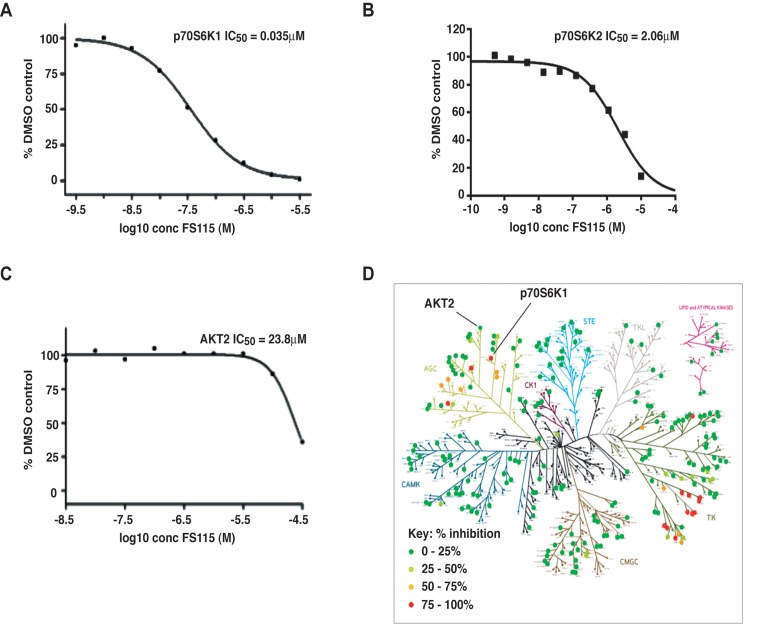
FS-115 inhibits p70S6K1 activity *in vitro* (**A**) p70S6K1 (**B**) p70S6K2 and (**C**) AKT2 were assayed in radiometric ATP-competitive kinase assay, in the presence or absence of increasing concentrations of FS-115 with 10mM Mg and 15μM ATP. The IC50 graph was plotted using XLFit version 5.3 (ID Business Solutions). Sigmoidal dose-response (variable slope) curves are fitted using non-linear regression analysis. The results are presented as the percentage of kinase activity relative to the DMSO-treated control. (**D**) Selectivity map displaying percentage inhibition of 268 kinases tested in radiometric ATP-competitive kinase assay, using FS-115 at 0.5μM. The extent of inhibition is represented by colored dots according to the scale displayed. Each dot represents the mean percentage inhibition derived from a duplicate experiment.

We recently published that inhibition of p70S6K1 activity strongly impaired local relapse in a mouse model of breast cancer recurrence [9]. In order to develop candidate drugs for clinical trials and translate our results from the bench to the bedside, we have tested a novel inhibitor of p70S6K1. We particularly looked to compounds that were potent, selective, cell penetrant and orally bioavailable. Among the different compounds, FS-115 exhibited particularly high selectivity and potency and was thus selected for further analyses. In particular, it blocked the kinase activity of p70S6K1, *in vitro*, with an IC50 of 0.035μM, as determined by using a radiometric ATP-competitive kinase assay (Fig. 1A). Importantly, it only marginally inhibited the activity of p70S6K2, a closely-related homologue of p70S6K1 (IC50 of 2.06μM) and AKT2 (IC50 of 23.8μM) (Fig. 1B-C). FS-115 also inhibited some tyrosine kinases of the Src family and PRK2 (protein kinase C-related protein kinase-2) and ROCK-II (Rho-associated protein kinase II), while it was substantially inactive towards other kinases of the PI3K pathway (Fig. 1D and Supplementary Table 1).

### FS-115 efficiently suppresses p70S6K activity in breast cancer cells

Our previous studies demonstrated that wound fluids, drained from BC patients for 24 hours post-surgery (hereafter WF), are very rich in cytokines and growth factors and very efficiently stimulate proliferation, motility and invasion and that activation of p70S6K signaling is a common event following exposure of breast cancer cells to WF [10,24].

**Figure 2:**
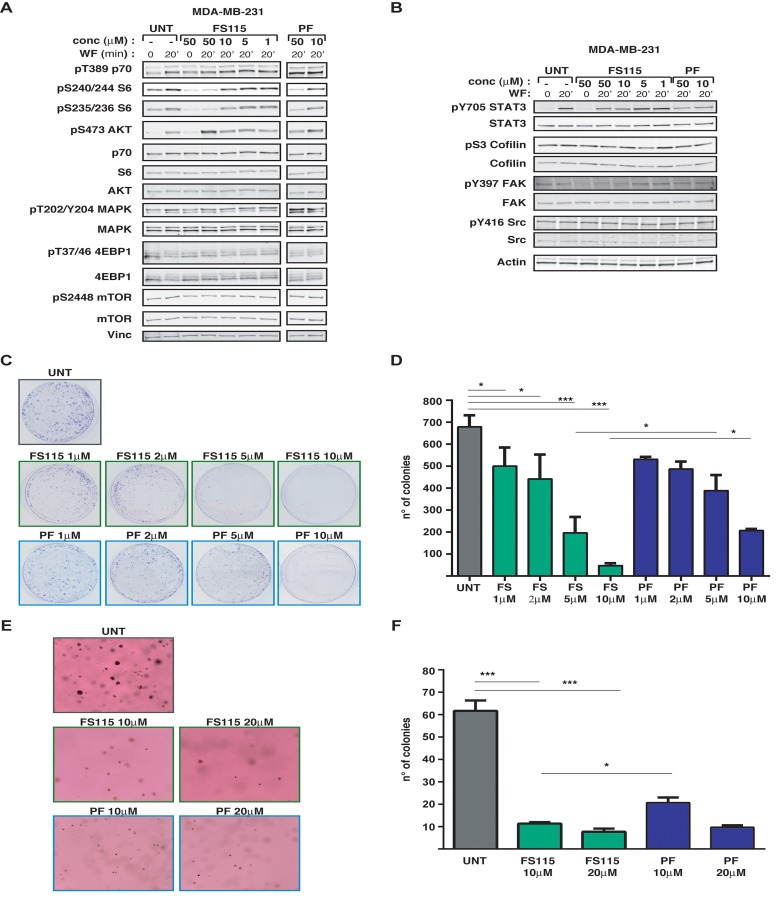
FS-115 efficiently suppresses p70S6K1 activity in MDA-MB-231 breast cancer cell line (**A**) Western blot analysis of MDA-MB-231 breast cancer cells, serum starved (time 0) and then stimulated for 20 minutes with wound fluids (WF) or serum starved, pre-treated 30 minutes with FS-115 or PF-4708671 (50-10-5-1μM, as indicated) then stimulated for 20 minutes with WF, always in the presence of the inhibitor. p70S6K, AKT, MAPK and mTOR signaling pathways were analyzed. Vinculin was used as loading control. (**B**) Same as in (**A**) but the analysis was focused on STAT3 and Src signaling pathways. Actin was used as loading control. (**C**) and (**D**) Colony assay of MDA MB 231 cells untreated (UNT) or pre-treated for 24 hours with FS-115 or PF-4708671 at the indicated concentration, then counted and seeded at ultra-low density (1000 cells/100 mm dish) and incubated in complete growth medium in the presence of the inhibitors or left untreated. Two weeks later plates were stained with crystal violet and colonies photographed (**C**) and counted (**D**). (**E**) and (**F**) Anchorage-independent cell growth of MDA-MB-231 cells included in soft agar in 3% wound fluids (UNT), in the presence of PF-4708671 or FS-115 at the indicate d concentration. After three weeks, colonies were photographed (**E**) and counted (**F**). Values reported in (**D**) and (**F**) represent the mean (± S.D.) of two independent experiments performed in duplicate. * p<0.05; ** p<0.01; *** p<0.001.

Thus, we tested the ability of FS-115 to counteract the potent activation of the p70S6K signaling induced in time course experiments of breast cancer cells stimulated with WF. To this aim, we serum starved MDA-MB-231 cells (triple negative subtype), pre-treated for 30 minutes with different doses of FS-115 and then stimulated with WF for 20 minutes, a condition in which p70S6K is highly activated [10]. Western Blot analyses of phosphorylated ribosomal protein S6 (pSer235/236- and pSer240/244-RPS6), the direct downstream substrate of p70S6K1, indicated that, following WF stimulation, FS-115 efficiently reduced p70S6K activity already when used at 10μM, at lower but comparable levels to what was observed using PF-4708671, a commercially available p70S6K inhibitor, used in most of the following experiments as control (Fig. 2A). Since it is known that prolonged treatment with mTOR inhibitors can lead to hyper activation of AKT, due to suppression of negative feedback loop mechanism [4], we checked the effect of FS-115 on this pathway. We noticed that FS-115 elicited a partial activation of AKT, but only when given at the highest dose (50μM). Similar results were harvested also in MDA-MB-453 (HER2-positive subtype) and MCF-7 (luminal subtype) breast cancer cells (Supplementary Figure 1).

Due to the relative *in vitro* activity displayed by FS-115 on c-Src, we also tested whether this signaling pathway was altered following treatment. We looked at c-Src expression and phosphorylation and, also, at the phosphorylation of STAT3, cofilin and FAK, three proteins frequently activated downstream of Src, in MDA-MB-231 cells stimulated with WF. However, no consistent effect of FS-115 on Src signaling pathway was detectable in MDA-MB-231 cells (Fig. 2B). In MDA-MB-453 c-Src expression and activation was barely detectable following WF stimulation and not affected by FS-115 (Supplementary Figure 1). On the other hand, in MCF-7 cells activation of c-Src was quite efficiently prevented by FS-115 treatment (Supplementary Figure 1), suggesting that the spectrum of activity for this inhibitor may vary depending from the cancer subtypes.

No effect on other members of p70S6K-related pathway (mTOR, 4EBP1) or on unrelated pathway (MAPK) was observed following FS-115 treatment (Fig. 2A).

Altogether, these experiments indicate that, following strong activating stimuli, FS-115 treatment efficiently and specifically suppresses p70S6K activity in breast cancer cells.

### FS-115 strongly impacts on the survival and tumorigenic potential of breast cancer cells

Our recent data suggested that an intact p70S6K signaling is important for cell survival, particularly when cells are challenged by “stringent” microenvironmental conditions. From previous studies, we know that MDA-MB-231 cells grow very well also in stringent culture conditions, such as plating at very low density, but robust p70S6K activity is necessary for their survival and for colony formation [10]. Colony formation assay on cells plated at a very low density (1×10^3^/100mm dish) demonstrated that treatment with FS-115 was highly potent in suppressing the ability of MDA-MB-231 cells to survive and form colonies in such condition, even when added at very low concentrations (Fig. 2C and D). Thus, FS-115 treatment was capable to suppress ability of breast cancer cells to survive to harsh environment, such as when they are challenged to grow as isolated colonies.

Next, we tested anchorage independent growth in soft agar, in the presence of WF as external source of growth stimuli, with or without FS-115 or PF-4708671, at different doses. Impairment of p70S6K activity, by either compounds, significantly decreased the cell ability to survive and grow in anchorage independence (Fig. 2E and F). Both number and size of the colonies were affected, signifying that, in this context, activity of p70S6K was necessary for both survival and proliferation of breast cancer cells (Fig. 2E and F). This result again supported that, under stringent conditions, cells rely on a robust p70S6K signaling for their survival and that FS-115 efficiently counteracts this process.

### Pharmacokinetic and pharmacodynamic profile of FS-115

Altogether, the above experiments supported that FS-115 was a promising compound, since it not only significantly and specifically blocked p70S6K activity but also well recapitulated our previous findings on the relevance of p70S6K activity in breast cancer cells. Given the good performance of FS-115 *in vitro*, we decided to evaluate the potential of this new compound *in vivo*.

**Figure 3:**
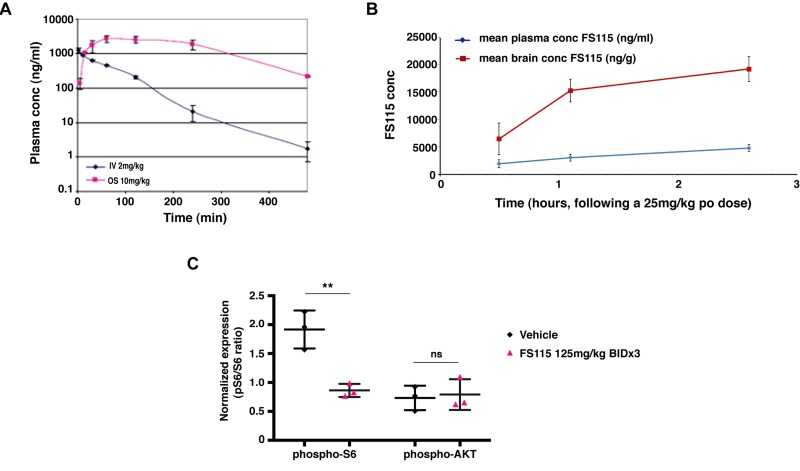
FS-115 shows excellent pharmacokinetic features (**A**) Graph reports the pharmacokinetic profile of FS-115 in male CD-1 mice, looking at plasma profiles following a single dose of FS-115, *via* both intravenous (IV, 2 mg/kg) and oral (OS, 10 mg/kg) routes of administration. Plasma was sampled at 8 time points (n=3 mice per time point, terminal sampling) over 24 hours. FS-115 was determined to exhibit an oral bioavailability in excess of 95%, an IV half-life of 0.83 hr, clearance of 25 ml/min/kg and a volume of distribution of 1.64 L. (**B**) Graph reports the brain-penetrance of FS-115 in male CD-1 mice, following a single dose of FS-115, given orally (p.o.) at 25 mg/kg. Plasma and brain were sampled at 3 time points (0.5, 1.1 and 2.6 hr). (**C**) Graph reports the pharmacodynamic profile of FS-115 in nude mice orthotopically implanted with MDA-MB-231 cells and treated with either vehicle QD x 3 PO or FS-115 125 mg/kg BID x 3 PO (6 doses in total). Twelve hours after the final dose animals were sacrificed, tumors resected and homogenized and ELISA for phospho-S6, total S6, phospho-AKT and total AKT. Data are normalized by the expression of the respective total protein. Differences were considered significant when p<0.05 (*) and calculated by two tailed t-test.

We first tested the pharmacokinetic profile of FS-115 in male CD-1 mice, looking at profiles from both intravenous (i.v.) and oral (p.o.) routes of administration. FS-115 was formulated using a solution comprised of 3% DMSO, 0.1M aqueous HCl (stoichiometric amount) and a 20% w/v aqueous solution of β-cyclodextrin (dose volume 5 ml/kg). Following a single dose of FS-115 at 10 mg/kg p.o. and 2 mg/kg i.v., plasma was sampled at 8 time points (n=3 mice per time point, terminal sampling) over 24 hours, followed by protein precipitation using methanol and analysis by LC-MS/MS. The plasma profiles are shown in Figure 3A. FS-115 was determined to exhibit an oral bioavailability in excess of 95%, an i.v. half-life of 0.83 hr, clearance of 25 ml/min/kg and a volume of distribution of 1.64 L. We next tested the brain-penetrance of FS-115 by a separate discrete oral pharmacokinetic experiment in male CD-1 mice. Following a single dose of FS-115 at 25 mg/kg p.o., plasma and brain was sampled at 3 time points (0.5, 1.1 and 2.6 hr), followed by protein precipitation using acetonitrile and analysis by LC-MS/MS. FS-115 was found to be highly brain-penetrant, with a ratio of (brain : plasma) of (4.0 : 1) at 2.6 hr (Fig. 3B).

For pharmacodynamic profile, female athymic nude mice were implanted with MDA-MB-231 cells into the fat pad of the thoracic mammary gland. When tumors reached 50-100 mm3, mice were randomly assigned to treatment groups of either vehicle QD x 3 PO or FS-115 125 mg/kg BID x 3 PO (6 doses in total). Twelve hours after the final dose animals were sacrificed, tumors resected and homogenized. Protein extracts were then tested in ELISA assay for the expression of total/phospho-S6 (ser 240/244) and total/phospho-AKT (ser 473). FS-115 treated animals exhibited significantly reduced expression of phospho-S6 compared to vehicle-treated animals (Fig. 3C). It is important to note that the effect of FS-115 was long lasting *in vivo*, the samples for ELISA having been taken 12 hours following the final dose. Furthermore, there was no effect on phospho-AKT, indicating that a pro-survival response had not been initiated by dosing of FS-115 (Fig. 3C).

### Treatment with FS-115 reduces tumor take rate and growth

**Figure 4:**
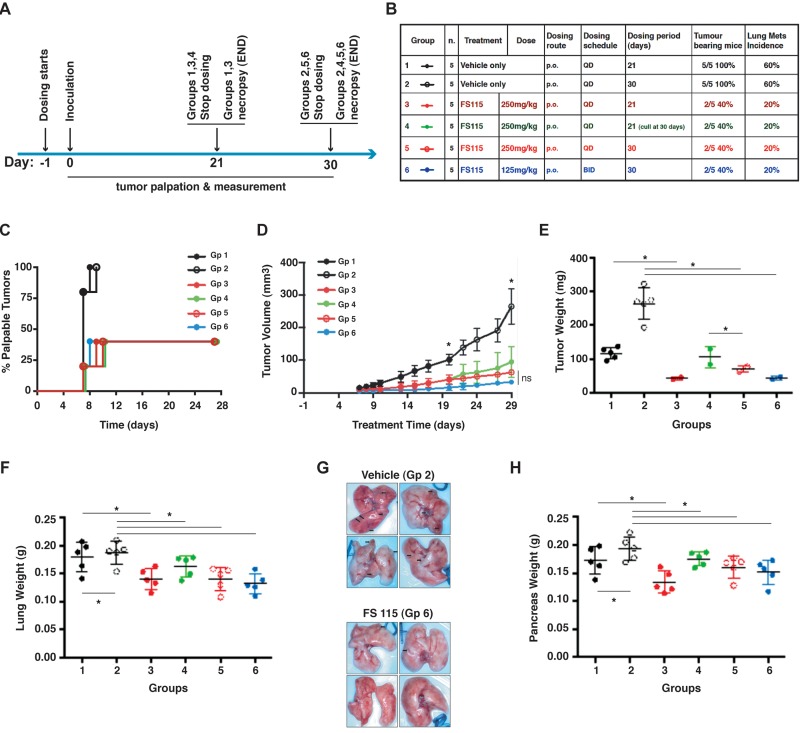
FS-115 reduces tumor take rate and growth (**A**) Schematic representation of the experimental workflow used for the tumor initiation experiment. (**B**) Table describes the different experimental groups in which mice were subdivided to test different FS-115 concentrations and different treatment schedules. Percentages of tumor bearing mice and lung metastasis formation in each group are also indicated. (**C**) Graph reports the time dependent appearance of palpable primary tumors derived from injection of MDA-MB-231 cells (1×10^6^), in the six groups treated as described in (**B**). (**D**) Same as in (**C**) except that tumor volume (mm^3^) was measured with a caliper. Animals from Group 1 (black line, solid circles) and those from Group 2 (black line, open circles) are combined in the same line until day 21, since they are all treated the same. At day 21, animals from Group 1 are culled, while those from Group 2 continue to be treated with FS-115 and measured, until day 30. The same is true for animals from Groups 3 (red line, solid circles) and Group 5 (red line, open circles). The asterisks at treatment day 20 and 29 indicate that, starting from these time points, there is significant difference (p<0.05) between vehicle (Group 2) and 250mg/kg QD x 30 (Group 5) and between vehicle (Group 2) and 125mg/kg BID x 30 (Group 6). (**E**) Same as in (**D**) except that tumor weight (g) was measured at necropsy (21 or 30 days from injection). (**F**) Same as in (**D**) except that lung weight (g) was measured at necropsy (21 or 30 days from injection). (**G**) Pictures show the lungs from two representative experimental groups (Group 2, Vehicle, QD, 30 days and Group 6, FS-115 125mg/kg, BID, 30 days). (**H**) Same as in (**D**) except that pancreas weight (g) was measured at necropsy (21 or 30 days from injection). In all graphs, * indicates a p<0.05. In all cases, differences were considered significant when p<0.05 (*) and calculated by two tailed t-test.

Based on these encouraging data, we moved to the evaluation of the effects of FS-115 in tumor initiation. To this aim, on day -1 animals were randomly assigned to treatment groups and drug treatment commenced. FS-115 was dosed orally daily (QD) at 250mg/kg or twice a day (BID) at 125mg/kg regimen, for 21 or 30 consecutive days (Fig. 4A and B). On day 0, MDA-MB-231 cells (1×10^6^ in Matrigel) were implanted into the fat pad of the thoracic mammary gland. Measurement of tumor volume was commenced 7 days following implantation. Treatment with FS-115 significantly reduced the take-rate of MDA-MB-231 tumors, with only 40% of animals having a palpable tumor at the end of the study. In contrast 100% of vehicle-treated controls had tumors (Fig. 4B and C). When tumors appeared, a significant reduction in tumor volume was apparent in all treated animals, already from day 9 after implantation (Fig. 4D, compare black line with any colored line from treated animals). Animals from Group 1 (black line, solid circles) converge in a same line with Group 2 (black line, open circles) until day 21, since they are equally treated and then culled. The same is true for animals from Groups 3 (red line, solid circles) and Group 5 (red line, open circles). Although not significantly, mean tumor volume of animals treated with 125mg/kg BID was consistently less than that of animals treated with 250mg/kg QD (Fig. 4D, compare blue and red lines). Group 4 animals (green line) received treatment for 21 days, followed by a recovery period of 7 days. It is interesting to note that during this recovery period tumor growth was faster than that observed in animals from Group 5 (red line) who continued to receive compound (Fig. 4D, compare green and red lines). However, mean tumor weight was significantly different between groups receiving vehicle *versus* each corresponding treatments (Fig. 4E).

Necropsy of vehicle-treated control animals revealed metastatic spread to lung and pancreatic tissues. Consistently, their lung tissue weighed significantly more than that collected from animals treated with FS-115 at 250mg/kg QD or 125mg/kg BID and animals sacrificed after receiving 30 doses of vehicle had lung tissue heavier than that from animals sacrificed 9 days earlier (mean weight 0.194g versus 0.17g) (Fig. 4F). Nodules on the surface of lungs were visible in 6/10 vehicle-treated animals (with a mean of 3 nodules per animal). In contrast, only 4/20 FS-115 treated animals had lung nodules with a mean of 1 nodule per animal (Fig. 4B and F). This pattern was also seen with pancreatic tissue (Fig. 4H). Liver weights were not significantly different (not shown). Thus, treatment with FS-115 reduced tumor initiation and spontaneous metastatic spreading of MDA-MB-231 cells to distant organs.

### Peri-surgical treatment with FS-115 suppresses breast cancer local recurrence

**Figure 5:**
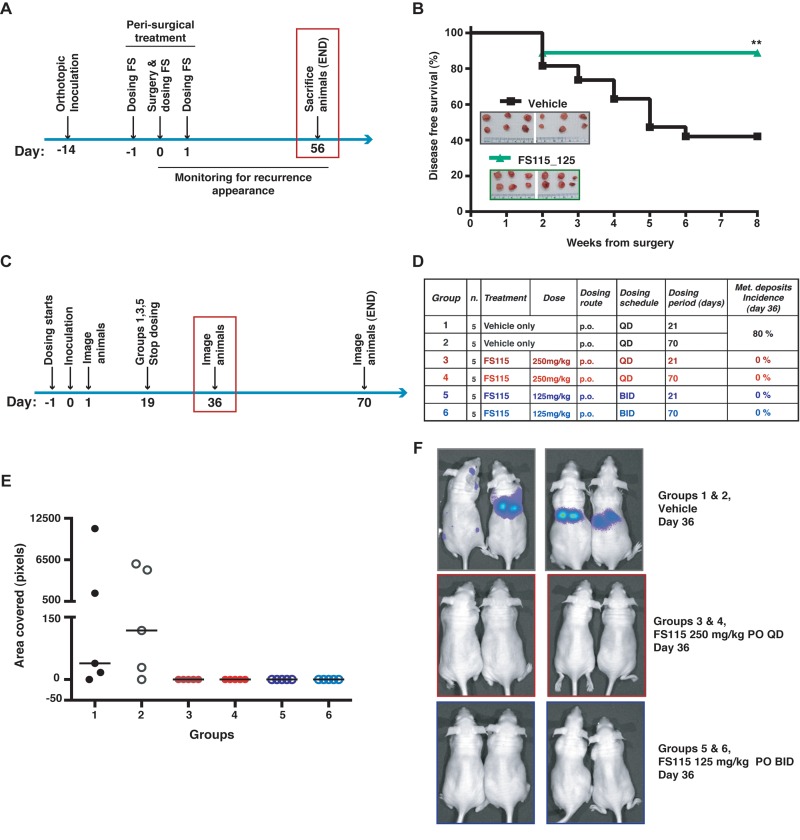
FS-115 suppresses breast cancer local recurrence and metastatic spread (**A**) Schematic representation of the experimental workflow used for the local recurrence formation experiment. (**B**) Graph reports the disease free survival curve of the mice injected with MDA-MB-231 cells (1×10^6^), and then peri-operatively treated with FS-115 (125mg/kg, BID) or vehicle. In the inset, pictures of the primary tumors collected the day of surgery (day 0) are shown. (**C**) Schematic representation of the experimental workflow used for the metastasis formation experiment. Mice were intracardiacally injected with MDA-MB-231-luc cells (1×10^5^) and imaged at the mid-point of study (36 days after inoculation). (**D**) Table describes the different experimental groups in which mice were subdivided to test different FS-115 concentrations and different treatment schedules. Percentage of metastatic deposits incidence, calculated at day 36 in each group, is also indicated. (**E**) Graph reports the quantification of the area covered by metastatic deposits (pixels), in the different experimental groups, imaged using a Xenogen IVIS machine, after injection with D-luciferin. (**F**) Pictures show two representative animals/group from the experiment described in (**C**), (**D**) and (**E**). In all cases, differences were considered significant when p<0.05 (*) and calculated by two tailed t-test. In all cases, differences were considered significant when p<0.05 (*) and calculated by two tailed t-test.

We next evaluated the efficacy of FS-115 in pre-clinical setting, using the mouse model of breast cancer recurrence recently set up in our laboratory [9,10]. Briefly, MDA-MB-231 breast cancer cells were bilaterally injected in nude mice mammary fat pads (MFP). Once grown, primary tumor masses were surgically removed and mice were followed-up for 8 weeks (Fig. 5A). Our previous studies demonstrated that in this context MDA-MB-231 breast cancer cells give rise to locally recurrent disease in 50-70% of cases and that impairment of p70S6K activity, by different means, strongly decreases the rate of recurrence formation of these cells [9,10]. We thus tested the activity of FS-115, treating mice with 125 mg/kg twice a day (BID) x 3, in a peri-surgical treatment schedule (administration at day -1, 0 and +1, respect to surgery time) (Fig. 5A). Although primary tumor masses had grown at similar extent (Fig. 5B, see insets), the evaluation of recurrence formation at long term (8 weeks) indicated that FS-115 was highly effective in reducing the rate of recurrence formation (Fig. 5B) (disease-free survival: 42% *vs* 84% in CTR *vs* FS-115-treated mice, p=0.02). Thus, this experiment supported that peri-surgical inhibition of p70S6K1 with FS-115 could represent a promising therapeutic agent to treat breast cancer patients at high risk to develop recurrent disease.

### Treatment with FS-115 strongly suppresses metastatic spread of breast cancer cells

**Figure 6:**
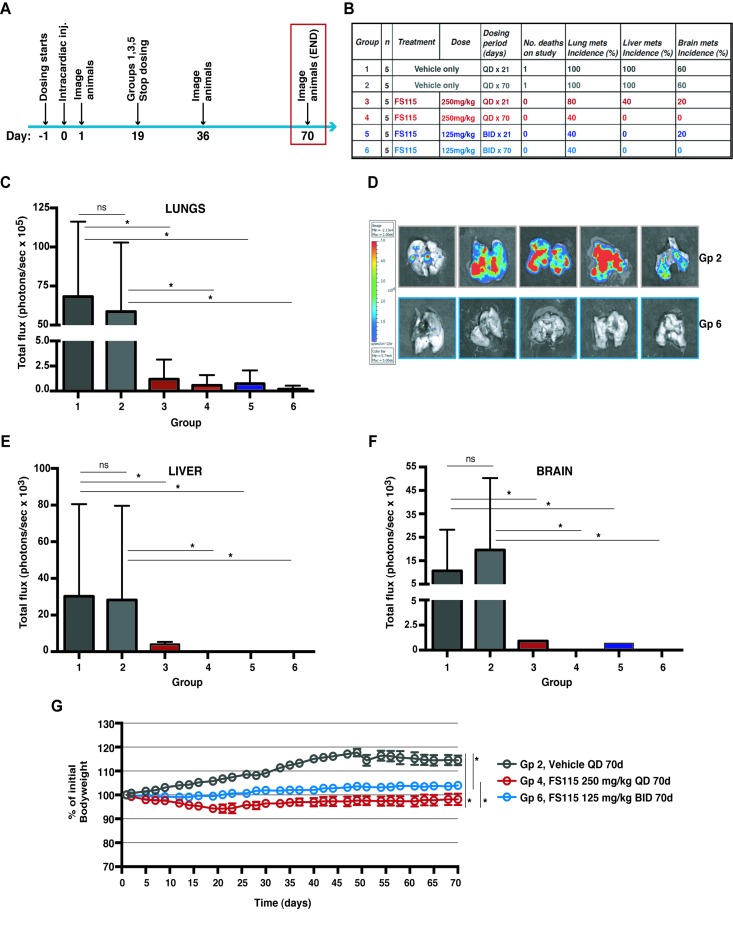
FS-115 is well tolerated and efficiently counteracts metastatic spread to distant organs (**A**) Schematic representation of the experimental workflow used for the metastasis formation experiment. Mice were intracardiacally injected with MDA-MB-231-luc cells (1×10^5^) and imaged at the end-point of study (70 days after inoculation). (**B**) Table describes the different experimental groups in which mice were subdivided to test different FS-115 concentrations and different treatment schedules. Number of death, percentages of lung, liver and brain metastasis incidence, calculated at day 70 in each group, are also indicated. (**C**) Graph reports the total flux (photons/sec) in the lungs extracted from mice of the different experimental groups. Mice were injected with D-luciferin before being culled, and then lungs imaged using a Xenogen IVIS machine. (**D**) Pictures show the lungs from two representative experimental groups (Group 2, Vehicle QD, 70 days and Group 6, FS-115 125mg/kg, BID, 70 days). (**E**) Same as in (C), except that livers were measured. (**F**) Same as in (C), except that brains were measured. (**G**) Graph reports the animal weight, expressed as percentage of initial bodyweight, in three representative experimental groups (Group 2, Vehicle QD, 70 days; Group 4 FS-115 250mg/kg, QD, 70 days; and Group 6, FS-115 125mg/kg, BID, 70 days). Bodyweight was annotated daily and used as a measure of drug tolerance. Group 4 is significantly different from Group 2 from 12 days of treatment. Group 6 is significantly different from Group 2 from 14 days of treatment. Group 4 is significantly different from Group 6 from 21 days of treatment. In all cases, differences were considered significant when p<0.05 (*) and calculated by two tailed t-test.

Finally, FS-115 was tested for efficacy in a mouse model of metastasis. Female athymic nude mice were intracardially inoculated with luciferase-tagged MDA-MB-231 cells (MDA-MB-231-Luc), to allow for evaluation and treatment of brain metastases. FS-115 was dosed orally QD at 250mg/kg or BID at 125mg/kg regimen, for 21 or 70 days, with dosing commencing the day prior to inoculation (Fig. 5C and D). At the mid-point of study (36 days after inoculation), 80% of vehicle-treated animals had visible metastatic deposits whereas none of compound-treated animals had visible metastatic deposits (Fig. 5E and F; Suppl. Fig. S2A). It is interesting to note that mice treated for 21 days (groups 3 and 5) showed no metastatic deposits, suggesting that a shorter duration of dosing may be sufficient to control metastasis. However, to better evaluate metastatic spread and longer term outcomes, all animals were imaged and sacrificed at day 70 (Fig. 6A and B). To this aim, brain, liver and lungs were removed and imaged individually (Fig. 6C and F; Suppl. Fig. S2B and C). Review of flux readings (which show severity of metastatic burden) showed that 70-days treatment regimen was most effective overall. Comparing groups 3 and 5, BID dosing was more effective than QD dosing and, FS-115 125 mg/kg BID x 70 (group 6) was the most effective regimen, with no metastasis detected in liver or brain and only minimal signal in lungs.

### Treatment with FS-115 is well tolerated in mice

Tolerability studies were performed in female athymic nude mice used for the experiments described above (Fig 6A), in which FS-115 was dosed orally QD at 250mg/kg or BID at 125mg/kg regimen, for 70 consecutive days. Bodyweight and general clinical signs were annotated daily and used as a measure of drug tolerance. No animals were sacrificed due to weight loss or illness, while 1 death in each of vehicle groups, due to metastatic burden, occurred (Fig. 6B). Treatment with FS-115 QD at 250mg/kg resulted in significantly decreased bodyweight respect to vehicle, however this never dropped below 10% of the initial (Fig. 6G). Dosing with 125mg/kg BID resulted in significantly less bodyweight loss than 250mg/kg QD (Fig. 6G), suggesting that this regimen might be better tolerated. The vehicle treated animals showed a decline in bodyweight post day 49 (probably concurrently with metastatic spread), while this was not observed for FS-115-treated animals (Fig. 6G). It is to consider that the slow weight gain observed in the FS-115-treated cohort is likely related also to the role of p70S6K1 in controlling organ size, as suggested by the phenotypes observed in p70S6K1 KO mice [25].

## DISCUSSION

The occurrence of locoregional recurrence in breast cancer patients strongly predicts the outcome of the disease. Disease progression into metastatic forms, after a highly variable period of clinical remission following treatment of the primary tumor, is responsible for most deaths from breast cancer. Little is known about the signaling pathways that permit residual cancer cells to survive and eventually re-grow. Thus, elucidating and successfully targeting the pathways underlying breast cancer recurrence is strongly needed in order to eradicate primary disease and achieve a better prognosis in patients.

p70S6K protein overexpression in breast cancer has been associated with increased risk of locoregional recurrence [20]. Our recent work corroborated this evidence and directly correlated the activity of p70S6K with breast cancer relapse [9]. We demonstrated that p70S6K activity is required for triggering the survival response in breast cancer cells “challenged” by harsh environments, such as the growth at very low density or in anchorage independence, *in vitro*, or in isolated form in the breast microenvironment, *in vivo* [9,10]. We observed that the peri-surgical treatment with p70S6K inhibitors, targeting residual breast cancer cells when conceivably they were more vulnerable, was sufficient to achieve a better locoregional disease control [9].

Given these promising pre-clinical results, we developed new p70S6K inhibitors that could meet the requirements of the clinics. In particular, we generated a new compound, FS-115, that together with potent inhibitory activity (IC50 35nM) displayed good pharmacokinetic and pharmacodynamic properties, was well tolerated, exhibited excellent oral bioavailability and was highly brain-penetrant. Treatment of breast cancer cells with FS-115 confirmed and corroborated our previous results, showing the relevance of p70S6K signaling pathway in supporting survival of isolated neoplastic cells. This finding was supported both in the peri-operative treatment with FS-115, proved to be an effective way to decrease the rate of local breast cancer recurrence, and in the long-term treatment regimen, proved to be very effective in abolishing the metastatic spread to distant organs, including brain.

The results that we collected in the tumor initiation experiment are at some extent in contrast with our previous report [10]. We previously observed that cells silenced for p70S6K or overexpressing a kinase dead mutant (p70KR), when implanted in MFP at high number, gave rise to palpable tumors in 100% of cases. Here, when FS-115 was administered *via* either QD (at 250mg/kg) or BID (at 125mg/kg) regimen, we observed a 40% of take rate. However, this discrepancy is probably due to the much higher target inhibition elicited by the daily or bi-daily treatment with FS-115, with respect to the inhibition obtained from silencing p70S6K or overexpressing the p70KR mutant in the cells prior to their injection.

Differently from inhibition of mTOR with derivatives of rapamycin, specific inhibition of p70S6K1 with FS-115 did not result in feedback activation of AKT signaling and of pro-survival response [4,6]. This is in line with our previous observation [9,10] and represents a crucial data for the potential clinical use of this drug.

Growth-promoting pathways, such as the mTOR pathway, are involved in both cancer and aging [26,27]. Accordingly, rapamycin and its derivatives prevent both age-related diseases and cancer in mammals, including humans [26,27]. When cell cycle is arrested, the growth signal (if it cannot reactivate cycling) drives senescence. It was shown that inhibition of mTOR precludes senescence (by inhibiting the so called geroconversion) and causes reversible quiescence [26–29]. These data are in accord with our previous observation that isolated breast cancer cells, residually left behind after surgery and Temsirolimus treatment in mice, are quiescent for a certain time and eventually re-grow, resulting in tumor recurrence [9]. However, in the presence of an arrested cell cycle, as for instance caused by p21 induction, isolated inhibition of p70S6K only partially inhibited geroconversion [30]. In our context, the FS-115 inhibitor was always administered in highly proliferating cells. Treatment with specific p70S6K inhibitors under this cellular context did not affect geroconversion, but rather induced apoptosis, in accord with our previous data [9]. Conceivably, the *in vivo* efficacy of FS-115 actually relies in this switch between a cytostatic to cytotoxic action, eventually leading to more efficient abrogation of tumor recurrence and metastasis formation.

A recent study, aimed to identify novel cancer treatments using poly-pharmacological approaches, strongly support the conclusions of our present work [31]. The authors use a Drosophila model of multiple endocrine neoplasia type 2 (MEN2) and a kinome-wide drug profiling. The genetic and chemical data indicate that an optimal drug for MEN2 would show activity against Ret, Src, S6K and Raf but limited activity against Tor. Notably, co-inhibition of S6K was required for optimal animal survival, whereas inhibition of Tor led to toxicity owing to release of negative feedback [31]. Recent data reported that dual Src and mTOR inhibition was highly effective in two mouse models of breast cancer [32]. It is to note that the Src family of kinases are, in addition to p70S6K1, among the few kinases inhibited by FS-115 and that c-Src expression has been identified as a potential target for the treatment of triple-negative breast cancer [33]. Although our data *in vitro* suggest that following stimulation with WF, c-Src signaling pathway is not significantly and consistently affected by FS-115, we cannot exclude that FS-115 could act *in vivo*, at least in part, with dual inhibitory activity. FS-115 also efficiently inhibited PRK2 (protein kinase C-related protein kinase-2) and ROCK-II (Rho-associated protein kinase II) (Fig. 1D and E), both previously implicated in triple negative breast cancer growth and invasion [34,35]. We cannot exclude that the effect on these two proteins, in addition to c-Src, may also contribute to the therapeutic efficacy of this drug.

Overall, our results suggest that p70S6K may represent a promising therapeutic target for suppressing recurrence, by providing, following surgery, efficient elimination of minimal residual disease. This is of particular importance since appearance of local recurrence does not only represent a very disturbing event to the patient but it is also harbinger of systemic disease recurrence, thus impacting on patients' survival [36]. It is noteworthy that inhibition of p70S6K with FS-115 also demonstrated high efficiency in suppressing metastatic spreading in organs, such as the brain, where other drugs have failed to achieve a sufficient concentration and, as a consequence, a therapeutic effect. In the future, it would be very interesting to characterize whether this effect is due to enhanced apoptosis of isolated cancer cells already settled at distant sites or whether it is mainly affecting the survival of circulating tumor cells (or both).

Altogether, our *in vitro* and *in vivo* data suggest that interfering with p70S6K activity with FS-115 in peri-surgical schedule or in long-term treatment regimen may represent reliable strategies to treat breast cancer patients at high risk to develop locoregional recurrence and distant organ metastasis.

## METHODS

### Study approval

Animal experiments on tumor local recurrence were reviewed and approved by the CRO of Aviano Institutional Organism For Animal Wellbeing (OPBA) and by the Italian Ministry of Health. Experiments were conducted according to that committee's guidelines. Metastasis, tumor initiation, PK and PD protocols used have been approved by the Axis Bioservices Animal Welfare and Ethical Review Committee, and all procedures were carried out under the guidelines of the Animal (Scientific Procedures) Act 1986. PK studies were performed via two separate external providers, Nikem (IT) and Pharmidex (UK), under approval and in accordance with the internal ethics policy in operation at the time of the study.

Wound Fluids (WF) from breast cancer patients were collected at CRO of Aviano. Scientific use of biological material was approved by the Ethics Committee of the CRO of Aviano, Italy. Specific informed consent was obtained from each patients.

### Kinase profile of FS-115

FS-115 ability to block p70S6K1 kinase activity was tested using radiometric ATP-competitive kinase assay (Eurofins Millipore KinaseProfiler™ Service Assay). The broader kinase selectivity profile was determined by counter-screening FS-115 against a panel of 268 kinases, excluding p70S6K1, using the Eurofins Millipore KinaseProfiler™ Service. Full details are available at http://www.eurofins.com/pharma-services/pharma-discovery-services/services/in-vitro-pharmacology/kinases.aspx. In brief, the kinase assay method used is as follows: FS-115 was prepared at 50x the final assay concentration in 100% DMSO. In a final reaction volume of 25 μL, human p70S6K1 protein of 1-421 amino acid length with T412E mutation is incubated with 8 mM MOPS pH 7.0, 0.2 mM EDTA, 100 μM KKRNRTLTV, 10 mM Mg acetate and [γ-33P-ATP] (specific activity approx. 500 cpm/pmol, concentration as required). After incubation for 40 minutes at room temperature, the reaction is stopped by the addition of 5 μL of a 3% phosphoric acid solution. 10 μL of the reaction mixture is then spotted onto a P30 filtermat and washed three times for 5 minutes in 75 mM phosphoric acid and once in methanol prior to drying and scintillation counting. The concentration of ATP used was 15 μM.

### Cell culture and development of stable cell lines

MDA-MB-231 (basal, ER-, PR-, HER2-), MDA-MB-453 (luminal, ER-, PR-, HER2-), MCF-7 (luminal, ER+, PR+, HER2-) mammary carcinoma cell lines were obtained from ATCC (LGC Standards) and grown in Dulbecco modified Eagle medium (DMEM, Lonza) supplemented with 10% fetal bovine serum (FBS, SIGMA). All cell lines were authenticated by BMR Genomics srl (Padova, Italy), according to Cell ID ^TM^ System (Promega) protocol and using Genemapper ID Ver 3.2.1, to identify DNA STR profiles. MDA-MB-231-luciferase-expressing cells (MDA-MB-231-luc) derived from MDA-MB-231 cells originally purchased from ATCC. For *in vivo* experiments, these cells were stably transfected with pGL4.51 (Promega) using Lipofectamine 2000 (Life Technologies), by Axis Bioservices (Northern Ireland, UK).

### Preparation of protein lysates and immunoblotting analysis

MDA-MB-231, MDA-MB-453, MCF-7 mammary carcinoma cell lines were serum starved in DMEM containing 0.1% bovine serum albumin (BSA, SIGMA) and then stimulated with 5% WF for 20 minutes. Where indicated, cells were also pre-treated for 30 minutes with PF-4708671 (p70S6K1 inhibitor, 10 or 50 μM, SIGMA) or FS115 (10 or 50 μM).

To extract total proteins cells were scraped on ice using cold RIPA lysis buffer (1% NP40; 50 mM Tris HCl pH 8; 150 mM NaCl; 0.1% SDS; 0.5% Na-Deoxycholate) plus a protease inhibitor cocktail (Complete™, Roche) and supplemented with 1 mM Na_3_VO_4_ (SIGMA), 10 mM NaF (SIGMA) and 1 mM DTT (SIGMA).

For immunoblotting analysis, proteins were separated in 4-20% SDS-PAGE (Criterion Precast Gel, Biorad) and transferred to nitrocellulose membranes (GE Healthcare). Membranes were blocked with 5% dried milk in TBS-0.1% Tween20 or in Odyssey Blocking Buffer (Licor, Biosciences) and incubated at 4°C overnight with primary antibodies. Then, membranes were incubated 1 hour at RT with IR-conjugated (Alexa Fluor 680, Invitrogen or IRDye 800, Rockland) secondary antibodies for infrared detection (Odyssey Infrared Detection System, Licor).

Primary antibodies directed against: AKT (sc-1618), ERK1 (sc-94), p70S6K1 (sc-8418), Src (sc-19), STAT3 (sc-482) and vinculin (sc-7694) were purchased from Santa Cruz; pT202/204 ERK1/2 (#9101), pS473 AKT (#4060), pT389 p70S6K1 (#9234), S6 (#2217), pS235/236 S6 (#4858), pS240/244 S6 (#5364), pS2448 mTOR (#2971), mTOR (#2972), pT37/46 4EBP1 (#2855), 4EBP1 (#9644), pY416 Src (#2101), pY705 STAT3 (#9131), pS3 Cofilin (#3311) were purchased from Cell Signaling; Cofilin (#612145), FAK (#610088) were purchased from BD Biosciences; pY397 FAK (#44624G) was purchased from ThermoFisher Scientific; Actin (#A5060) was purchased from SIGMA.

Wound fluid collection. Drainage wound fluids (WF) were collected over the 24hrs after surgery from unselected patients undergone breast-conserving surgery, as described previously [24,37]. The assays were then performed using pools of all fluids.

### Colony assay

For colony assay, MDA-MB-231 untreated or pre-treated for 24 hours with PF-4708671 (p70S6K1 inhibitor, 1, 2, 5, 10 μM, SIGMA) or FS-115 (1, 2, 5, 10 μM) were trypsinized, counted and seeded at density 1000 cells/100 mm dish and incubated in complete growth medium in the presence of the inhibitors or left untreated. Two weeks later plates were stained with crystal violet and colonies were counted.

### Soft Agar assay

To evaluate the anchorage-independent cell growth, MDA-MB-231 cells (1.5×10^4^) were re-suspended in 2 ml top agar medium (DMEM-10% FBS or SFM-3% WF, 0.4% Low Melting Agarose, SIGMA) in the presence of PF-4708671 (p70S6K1 inhibitor, 10 or 20μM, SIGMA) or FS-115 (10 or 20μM), and quickly overlaid on a previously gelified 0.6% bottom agar medium (DMEM-0.1% BSA, 0.6% LowMelting Agarose, SIGMA). The experiments were performed in six-well tissue culture plates, in triplicate. Fresh medium was added to the wells twice a week as a feeder layer. After three weeks, the number of colonies was counted at 4X magnification.

### In vivo experiments

Pharmacokinetics (PK) studies were conducted by Nikem (IT) and Pharmidex (UK), in male CD-1 mice, looking at profiles from both intravenous (i.v.) and oral (p.o.) routes of administration. FS-115 was formulated using a solution comprised of 3% DMSO, 0.1M aqueous HCl (stoichiometric amount) and a 20% w/v aqueous solution of β-cyclodextrin (dose volume 5 ml/kg). Following a single dose of FS-115 at 10 mg/kg p.o. and 2 mg/kg i.v., plasma was sampled at 8 time points (n=3 mice per time point, terminal sampling) over 24 hours, followed by protein precipitation using methanol and analysis by LC-MS/MS. Brain-penetrance of FS-115 was determined by a separate discrete oral PK experiment in male CD-1 mice. Following a single dose of FS-115 at 25mg/kg p.o., plasma and brain was sampled at 3 time points (0.5, 1.1 and 2.6 hr), followed by protein precipitation using acetonitrile and analysis by LC-MS/MS.

Pharmacodynamics (PD) was conducted by Axis Bioservices in 6 female athymic nude mice (Hsd:Athymic Nude-Foxn1^nu^) implanted with MDA-MB-231 cells into the thoracic mammary fat pad (1×10^6^ in Matrigel). The mice were randomly assigned to treatment groups of either vehicle QD x 3 PO or FS-115 125 mg/kg BID x 3 PO (6 doses in total) when their tumors reached 50-100 mm3. Vehicle used for both treatment groups was 5% DMSO, 95% aqueous hydroxypropyl-β-cyclodextrin (20% w/v). Twelve hours after the final dose animals were sacrificed and the tumor resected. Tumor tissue was homogenized in three volumes Pathscan Sandwich ELISA lysis buffer (Cell Signaling Technologies). Protein concentration was measured using a commercial BCA kit (Sigma) and ELISAs for phospho-S6 240/244, total-S6, phospho-AKT 473 and total-AKT carried out according to manufacturer's instructions (Cell Signaling Technologies).

For local recurrence studies, primary tumors were established by injection of 2×10^6^ MDA-MB-231 control bilaterally in the fat pads of the thoracic mammary glands of female nude mice (Hsd:Athymic Nude-Foxn1nu, aged 6-8 weeks, Harlan). After the appearance of palpable primary tumors, animals were orally treated with FS-115 (125mg/kg) twice a day (BID) in a peri-surgical treatment schedule (day -1, day 0 and day +1 respect to surgery). Mice were sacrificed at the end point of the experiment (8 weeks from surgery) unless they displayed suffering behavior, in adherence with ethical guidelines of CRO of Aviano Institutional Organism For Animal Wellbeing (OPBA). Tumors, recurrences, lymphnodes and other organs were collected and stored for subsequent analyses.

Tumor initiation, tumor growth and metastasis studies were conducted by Axis Bioservices (Northern Ireland, UK) in female athymic nude mice (Hsd:Athymic Nude-Foxn1^nu^), aged 6-8 weeks.

For metastasis study, animals were initially assigned to treatment groups. On day 0, 1×10^5^ MDA-MB-231-luc cells were diluted in 100μl HBSS and loaded into a 27-gauge needle for intracardiac injection. The following day, animals were first injected with D-luciferin (i.p. at 150mg/kg) and then imaged using a Xenogen IVIS machine. Evidence of light only in the cardiac area is indicative of a failed implantation and so any such animals were discarded from the study. FS-115 (or vehicle) was dosed orally *via* QD (at 250mg/kg) or BID (at 125mg/kg) regimen, with dosing commencing the day prior to inoculation (day -1) and ending 70 days after inoculation. Animals were imaged on 2 occasions during the study, on day 38 from commencement of dosing (mid-point) and on day 70 (end of study).

For tumor initiation and growth studies, female nude mice were randomly assigned to treatment groups: FS-115 (or vehicle) was dosed orally *via* QD (at 250mg/kg) or BID (at 125mg/kg) regimen, with dosing commencing the day prior to inoculation (day -1) and ending 21 or 30 days after cell implantation. MDA-MB-231 cells (1×10^6^ in matrigel) were implanted into the second mammary fat pad, on day 0. Time to appearance and tumor volume were estimated respectively by palpation and caliper measurements.

In all *in vivo* experiments, dosing volume of FS-115 was 10 ml/kg and formulation was 5% DMSO, 95% aqueous hydroxypropyl-β-cyclodextrin (20% w/v). Dosing formulation was made up fresh daily. Bodyweight and general clinical signs (*e.g*. starred fur, lack of movement, difficulty breathing) were annotated daily and used as a measure of drug tolerance.

### Statistical analysis

The IC50 graphs were plotted using XLFit version 5.3 (ID Business Solutions). Sigmoidal dose-response (variable slope) curves are fitted using non-linear regression analysis. The results are presented as the percentage of kinase activity relative to the DMSO-treated control. Data were examined using the two-tailed Student t test or unpaired two-tailed Mann-Whitney U test. Differences were considered significant at p < 0.05. The computer software PRISM (version 4, GraphPad, Inc.) was used to make graphs and all statistical analyses, unless otherwise specified.

## SUPPLEMENTARY DATA


