# Supplementary material for: Preclinical validation of a novel compound targeting p70S6 kinase in breast cancer
Source: Aging (Albany NY). 2016 May 4;8(5):958–73. doi: 10.18632/aging.100954 (PMC4931847; doi:10.18632/aging.100954)
Supplement: Supplementary file 1 [file aging-08-0958-s001.pdf]

SUPPLEMENTARY DATA

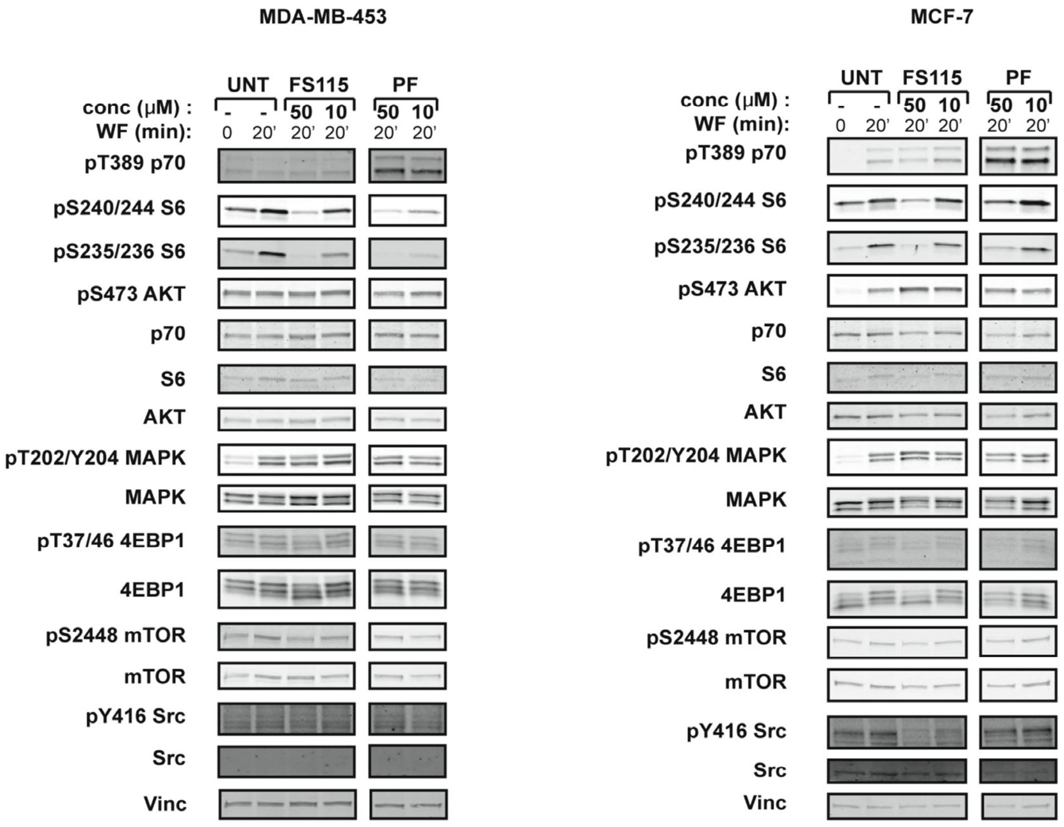

Supplementary Figure S1. FS-115 inhibits p70S6K1 in different breast cancer cell lines. (**A**) Western blot analysis of MDA-MB-453 breast cancer cells, serum starved (time 0) and then stimulated for 20 minutes with wound fluids (WF) or serum starved, pre-treated 30 minutes with FS-115 or PF-4708671 (50-10-5-1µM, as indicated) then stimulated for 20 minutes with WF, always in the presence of the inhibitor. p70S6K, AKT, MAPK, mTOR and Src signalling pathways were analyzed. Vinculin was used as loading control. (**B**) Same as in (**A**) but in MCF-7 breast cancer cells.

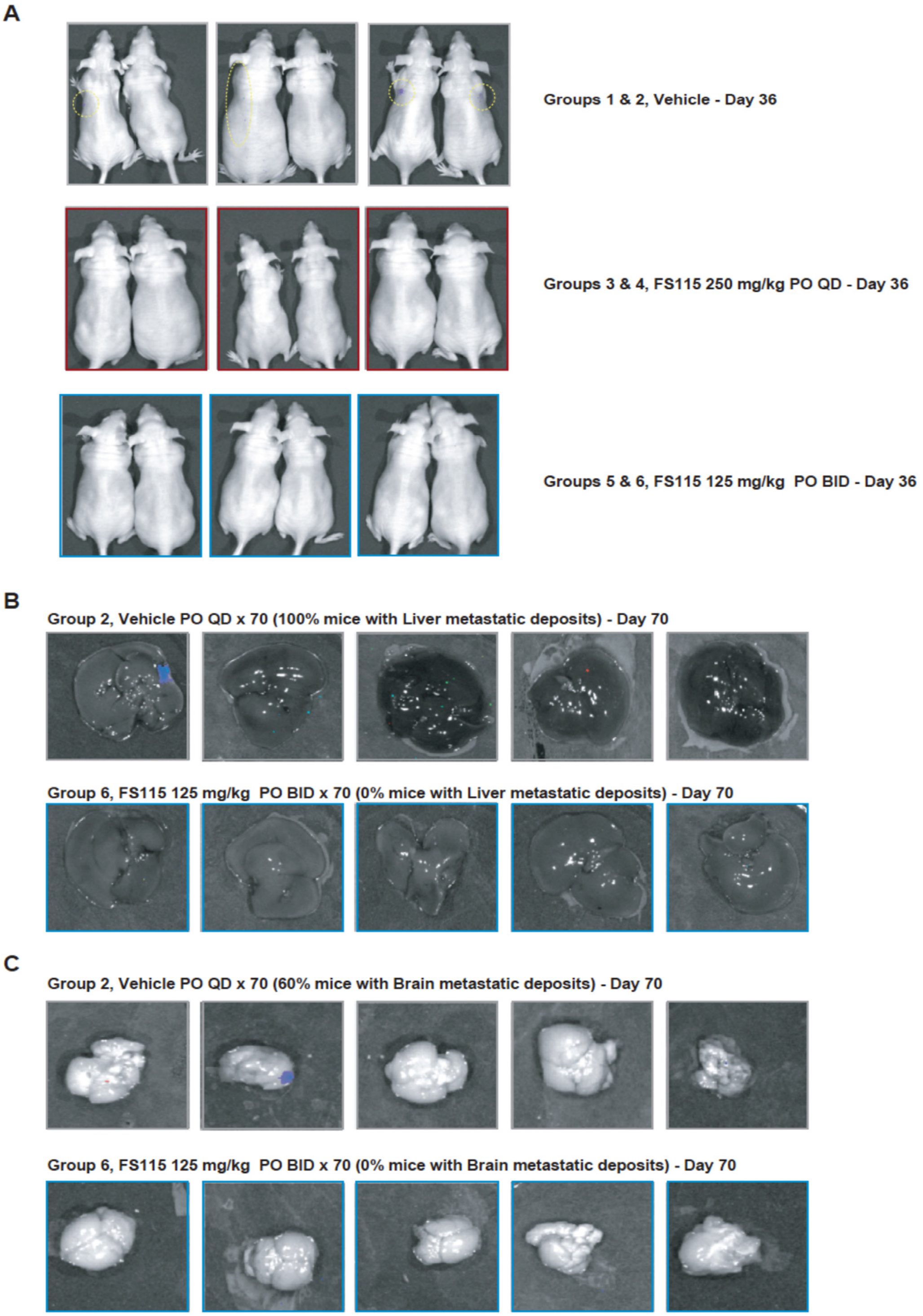

Supplementary Figure S2. FS-115 efficiently counteracts metastatic spread to distant organs. (**A**) Pictures show all animals from the experiment described in Figure 5 (except those reported in Figure 5), imaged using a Xenogen IVIS machine, after injection with D-luciferin, at day 36 from injection. (**B**) Pictures show livers from mice of two representative groups (Group 2, Vehicle QD, 70 days; and Group 6, FS-115 125mg/kg, BID, 70 days), from the experiment described in Figure 6. Mice were injected with D-luciferin before being culled, and then livers imaged using a Xenogen IVIS machine. (**C**) Same as in (**B**) except that brains were imaged.

KINASE	IC50 (μM)	% inhib. at 0.5 μM ^
p70S6K1	0.035	92
p70S6K2	2.06	11
Akt2	23.8	0
AMPKα1		10
AMPKα2		11
eEF-2K		0
MSK1		75
mTOR		11
NEK6		7
PDK1		3
PI3K (p110β/p85α)		1
PKA		67
PKCα		1
PRK2		93
ROCK-II		95
Rsk1		34
Rsk2		0
SGK		18
cSrc		89

Supplementary Table S1. Inhibition of key kinases by FS-115. Table displays the IC50 (μM) and the percentage of inhibition of the indicated kinases, from the 268 tested in radiometric ATP-competitive kinase assay, using FS-115 at 0.5μM. Percentage inhibition represents the mean derived from a duplicate experiment.
